# Effects of *Phaseolus vulgaris* Extract on Lipolytic Activity and Differentiation of 3T3-L1 Preadipocytes into Mature Adipocytes: A Strategy to Prevent Obesity

**DOI:** 10.1155/2019/5093654

**Published:** 2019-04-28

**Authors:** Felipe Castillo, Daniel R. González, Rodrigo Moore-Carrasco

**Affiliations:** ^1^Departamento de Bioquímica Clínica e Inmunohematología, Facultad de Ciencias de la Salud, Universidad de Talca, Talca, Chile; ^2^Laboratorio Clínico, Clínica Alemana de Santiago, Facultad de Medicina Clínica Alemana, Universidad del Desarrollo, Santiago, Chile; ^3^Departamento de Ciencias Básicas Biomédicas, Facultad de Ciencias de la Salud, Universidad de Talca, Talca, Chile; ^4^Programa de Investigación Interdisciplinario de Excelencia en Quimica y Bio‐Organica de Recursos Naturales (PIEI‐QUIM‐BIO), Universidad de Talca, Talca, Chile; ^5^Centro de Estudios en Alimentos Procesados (CEAP), CONICYT-Regional, Gore Maule R09I2001, Chile; ^6^Programa de Investigación Asociativa en Cáncer Gástrico (PIA-CG), Universidad de Talca, Talca, Chile

## Abstract

**Background:**

Overweight and obesity are defined as abnormal or excessive fat accumulation that may be harmful for health. A global trend in this area is the search for natural compounds that have a proven beneficial effect and no clinical complications. *Phaseolus vulgaris* (bean) is a vegetable highly consumed worldwide. One of its effects, the most reported, is weight reduction in overweight individuals.

**Objective:**

The objective of this study was to investigate the antiobesity activity of this legume in mature 3T3-L1 adipocytes and in rat white adipose tissue in an *ex vivo* model.

**Design:**

Mature adipocytes 3T3-L1 and rat adipose tissue were treated with bean extracts. We quantified lipolysis in mature 3T3-L1 adipocytes and in rat white adipose tissue in an *ex vivo* model.

**Results:**

In an *ex vivo* assay with adipose tissue, methanolic and aqueous green bean extracts increased glycerol release to the medium compared to control (*p* < 0.05 and *p* < 0.001 respectively). Treatment of 3T3-L1 adipocytes with green bean extracts (800 and 1000 *µ*g/mL) increased glycerol release significantly (*p* < 0.0001). Extracts at concentrations between 500 and 1000 *µ*g/mL reduced intracellular triglyceride accumulation by 34.4% and 47.1% compared to control (*p* < 0.0001).

**Discussion:**

Our results propose that bioactive compounds of green beans exert a direct mechanism on adipocytes through lipolysis.

**Conclusion:**

We have identified a novel capacity of bean extracts related to lipolytic activity both *in vitro* and *ex vivo*, resulting in a powerful lipolytic effect. Moreover, we also found that bean extracts has an antiadipogenic effect during the differentiation of 3T3-L1 preadipocytes. These results suggest that bean is a good candidate for the development of functional ingredients that can help reduce the high rates of death from cardiovascular diseases associated with obesity.

## 1. Introduction

Obesity is defined as having a body mass index greater than 30 kg/m^2^ and has been recognized as a global epidemic with a myriad of detrimental health effects [1]. For this reason, overweight and obesity are currently a health problem worldwide. It is estimated that the number of overweight and obese individuals has increased from 921 million in 1980 to 2.1 billion in 2013 [2]. Excessive body fat, especially higher visceral adiposity, is associated with an increased risk in the development of numerous adverse health conditions including diabetes and cardiovascular disease [3, 4].

This disorder is characterized by enlargement of adipose tissue, which results from the multiplication of fat cells followed by adipogenesis and increased deposition of cytoplasmic triglycerides [5]. It therefore corresponds to an increased number and size of adipocytes differentiated from fibroblastic preadipocyte precursors [6].

The process of adipogenesis of 3T3-L1 cells into mature adipocytes involves a highly orchestrated series of events including clonal expansion, growth arrest, and terminal differentiation [7]. Several *in vitro* models, particularly murine 3T3-L1 preadipocytes which can be differentiated into mature adipocytes, have improved our understanding of the mechanisms involved in obesity [8, 9]. Inhibition of adipogenesis and restoration of adipocyte function are considered to be important antiobesity mechanisms.

In the literature, it has been reported a large number of natural products that are capable of inhibiting adipogenesis, to induce apoptosis of adipocytes and/or to stimulate lipolysis. This would have great potential for treating and preventing obesity [10, 11].

Among the main foods that have these characteristics and are consumed by people worldwide are legumes. Within the group of leguminous plants that have edible seeds, beans or common beans (*Phaseolus vulgaris*) correspond to one of the most consumed. It is currently distributed in the five continents and is an essential component of the diet, especially in Central and South America where it is consumed as dry grain and green beans [12].

Previous studies have shown the nutritional and beneficial effects on metabolism of beans consumption, showing a wide range of phytochemicals, many of them with antioxidant capacity *in vitro* [13]. It has been shown that intake of beans exerts inhibitory effects on appetite as well as beneficial effects on carbohydrate metabolism both in rodents and in humans [14, 15]. Also, those common beans are nutritional ingredients that reduce the risk of cardiovascular diseases associated with platelet hyper-reactivity [16].

This effect on carbohydrate metabolism is produced by a group of inhibitors of the activity of enzymes responsible for degradation of complex carbohydrates from the diet, preventing their absorption [17]. Gupta et al. purified a potent inhibitor present in *Phaseolus vulgaris* with the ability to inhibit the activity of human salivary alpha-amylase [18]. This effect on carbohydrate metabolism has been assumed to be related to weight loss in humans and animals. However, this effect alone could not explain all the observed effects observed *in vivo*.

The aim of the present study was to investigate the antiobesity activity of this legume by quantifying lipolysis in mature 3T3-L1 adipocytes and in rat white adipose tissue in an *ex vivo* model.

## 2. Materials and Methods

### 2.1. Bean Samples

To evaluate the *ex vivo* lipolytic effect, we used the variety *of Phaseolus vulgaris* (bean) at different growing seasons (green beans (green pods and grain) and fresh beans (shelled bean)) for study. For the *in vitro* lipolytic and antiadipogenic effect, we only used green beans. The samples were selected and obtained from the Regional Supply Center (CREA), Talca, Chile.

### 2.2. Aqueous Extracts

Selected beans were washed and cut into small pieces. Using a blender were crushed and then methanol was added (Sigma-Aldrich, St. Louis MO, USA) in a ratio 80 : 20 distilled water/methanol. Then, the mixture was sonicated (Transsonic 700/H, Elma-Hans Schmidbauer, Germany) for 15 minutes, and then filtered with filter paper twice. The filtrate was subjected to rotary evaporation (RE 111-B461, BÜCHI Labortechnik AG, The Netherlands) for the full elimination of methanol. The resulting liquid was lyophilized (Freezone 6 Labconco, USA) and then was weighed and stored until use at −70°C (Ultra Low, Sanyo Electric Co., Ltd., Japan).

### 2.3. Animals

The samples used were obtained from dorsal white adipose tissue, from male Sprague–Dawley rats (obtained from the animal facility of Universidad de Talca) weighing between 200 and 300 g. The animals were maintained at 22 ± 2°C with a regular light-dark cycle (12 hour light and 12 hour dark) and had free access to food and water. All animal manipulations were made in accordance with the Bioethical Committee of the National Commission of Science and Technology, CONICYT, Chile, and approved to the Bioethical Committee of the University of Talca. For adipose tissue extractions, the abdominal cavity of each rat was opened, the intestines were removed, and the area beside the vertebral behind the kidneys spine was exposed. Then, adipose tissue was removed and washed three times with cold PBS. Subsequently, the extracted tissue was divided into segments of 100–110 mg.

### 2.4. Anesthesia and Sacrifice

Animals were weighed and anesthetized with a ketamine (50 mg/kg) (anesthetic)/xylazine (5 mg/kg) (muscle relaxant)/acetopromazine (1 mg/kg) (sedative). Death was caused by blood collection after the opening of the peritoneal cavity and secured by diaphragm rupture. The surgical material used was sterile.

### 2.5. Continuous Gassing System

We used a custom-made gas distributor of carbogen from a source of carbogen (95% O_2_-5% CO_2_) to a series of glass vials which contained 4 mL Krebs-HEPES buffer (pH 7.4 (reaction medium)) and white adipose tissue. Glass vials were immersed in a temperature-controlled bath under constant stirring at 37°C when gassing with carbogen.

### 2.6. *Ex Vivo* Lipolysis in WAT

Each glass vial was filled with 4 ml of buffer containing 1 mg/ml green bean extract and 1 *μ*M epinephrine (Sigma-Aldrich) as a positive control of adipose tissue lipolysis, via their action on the *β*2‐adrenergic receptor. Vials were incubated for 15 minutes at 37°C. After 15 minutes of preincubation, the WAT pieces were added into the vials and were incubated for 1 hour at 37°C under constant stirring. After incubation, the continuous gassing system was stopped and the vials were placed on the ice to terminate the reaction. The adipose tissue was removed from the liquid, and the medium was stored at −80°C until measurement of glycerol.

### 2.7. Cell Culture and Adipocyte Differentiation

3T3-L1 mouse preadipocyte cell line was obtained from American Type Culture Collection (ATCC, Manassas, VA, USA) and cultured in Dulbecco's modified Eagle medium (DMEM) high glucose (HyClone Laboratories, USA, Cat No. SH30243.01) containing 10% fetal bovine serum (HyClone Laboratories, USA, Cat No. SH30910.03) and 100 U/mL penicillin-streptomycin (Biological Industries, USA, No. 03-033-1B) at 37°C in a humidified 5% CO_2_ incubator. Cells were seeded in 12-well plates at a density of 3 × 10^5^ cells/well or in 24-well plates at a density of 6 × 10^4^ cells/well. Cells were grown to confluence 90% in DMEM/high glucose containing 10% FBS at 37°C and 5% CO_2_ in humidified air. Forty-eight hours after visual confluence (day 0), cell differentiation was induced by culturing in adipogénesis-inducing medium (DMEM/high glucose containing 10% FBS, 1 *µ*M dexamethasone (Sigma, USA, Prod. Nos. D4902), 0.5 mM IBMX (Sigma, USA, Prod. Nos. I5879), and 10 *μ*g/mL insulin (Sigma, USA, Prod. Nos. I927)) for two days. On Day 2, cells were then cultured in adipogenesis maturation medium (DMEM/high glucose containing 10% FBS and 10 *μ*g/mL insulin) for two days. Subsequently the cells were cultured in maintenance medium (DMEM/high glucose 10% FBS) for six days, with refreshment every two days. The effect on adipogenesis was assessed by adding increasing concentrations of the plant extract (500, 800, and 1000 *µ*g/mL) to the culture media during all processes of differentiation. Cells cultured only in maintenance medium were used as a control in all assays.

### 2.8. Cytotoxicity Assay

3T3-L1 preadipocytes were seeded in 96-well plates (2 × 10^4^ cells/well) and allowed to adhere overnight in DMEM/high glucose. After discarding the medium, a culture medium containing green bean extracts (20 to 1000 *µ*g/mL green beans) was added to each well and the cells were incubated for 24 to 48 h; untreated cells were used as controls. After completion of the time period, the cells were washed with PBS carefully and the medium was replaced with a medium containing MTT (3-[4,5-dimethylthiazol-2-yl]-2,5-diphenyltetrazolium bromide) 5 mg/ml (10 *µ*L/well) in DMEM and incubated at 37°C for an additional 4 h. Finally, the medium was removed, and the formazan produced was dissolved in 100 *µ*L HCl 0.1 N solution in isopropanol for 30 minutes in darkness. Absorbance of solution was measured at 570 nm using a microplate reader (Multiskan Go, Thermo Scientific). Cell proliferation (%) was calculated by the following equation: (absorbance of the sample/mean absorbance of the control) × 100.

### 2.9. *In Vitro* Lipolysis in 3T3-L1 Cells

The effect of the green bean extracts on lipolysis was quantified by adding increasing concentrations (20, 40, 60, 500, 800, and 1000 *µ*g/ml) of green bean extract to differentiated adipocytes for 48 h. Isoproterenol (Sigma, USA, Lot SLBK3425V), a known stimulator of adipolysis, was used as a positive control in all assays. Glycerol release was quantified using a kit (EnzyChrom™ Glycerol Assay Kit, BioAssay Systems, USA).

### 2.10. Oil Red O Staining

3T3-L1 preadipocytes were differentiated in 24-well plates and treated with the extracts as described previously. After differentiation, cells were washed twice with PBS and fixed with 4% (v/v) paraformaldehyde for 1 h at room temperature. Thereafter, cells were washed one time with PBS and one more time with isopropanol 60% (v/v) and were allowed to dry. Then, cells were stained with filtered Oil Red O solution 0.5% (v/v) (60% isopropanol and 40% water) for 1 h. After staining, the Oil Red O staining solution was removed, and the plates were rinsed with distilled water thrice and dried. The stained lipid droplets were viewed at 20*x* and 40*x* magnification on a microscope and were photographed.

### 2.11. Intracellular Lipids Accumulation

The stained oil droplets were solubilized by incubating with isopropanol 100% (v/v) for 15 min and absorbance, an indication of lipid accumulation, was quantified at 492 nm on a plate reader (Multiskan Go, Thermo Scientific).

### 2.12. Triglyceride Assay

To analyze the content of cellular triglycerides, after differentiation and treatment with extracts in 24-well plates, 3T3-L1 adipocytes were washed with PBS, scraped into 200 *µ*l PBS, and sonicated for 3 min. Thereafter, the suspension was centrifuged to 3500 rpm for 10 min. Intracellular triglycerides were quantified using a triglyceride kit according to the manufacturer's instructions (Triglycerides Liquicolor, Human, Germany). The results were expressed as % of triglycerides compared to control.

### 2.13. Statistical Analysis

Data are expressed as mean ± standard error of the mean (SEM). Differences between groups were analyzed by one-way analysis of variance (ANOVA) using Tukey's post hoc test using SPSS version 17.0 (SPSS, Inc., Chicago, Illinois) and GraphPad Prism 7. A value of *p* < 0.05 was considered significant.

## 3. Results

### 3.1. Effects of Bean Extracts on Lipolysis *Ex Vivo*

To assess the ability of bean extracts to stimulate lipolysis under physiological conditions, a series of *ex vivo* assays with rat adipose tissue was performed. The dorsal adipose tissue was removed from SD rats; this was washed with saline and incubated in the presence of different extracts at 1 mg/ml concentration. Lipolysis was assessed by measuring glycerol released to the medium as a marker for degradation of triglycerides.

**Figure 1:**
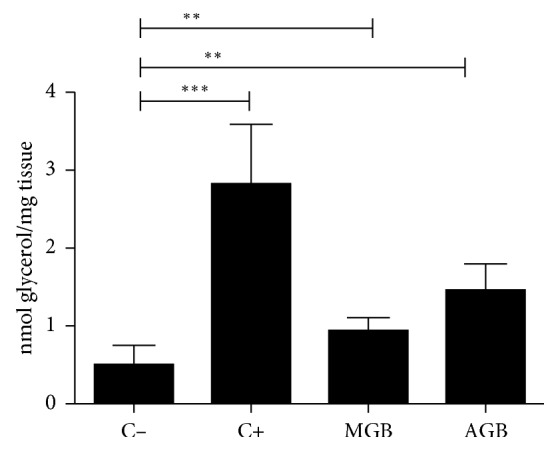
Effect of methanol extracts of green beans (MGB) and aqueous green beans (AGB) on lipolysis *ex vivo*. C−: negative control; C+: positive control; *n*=5. ^*∗∗*^*p* < 0.05; ^*∗∗∗*^*p* < 0.001.

Figure 1 shows the results obtained for aqueous and methanolic extracts of green beans, where it is appreciated that both extracts have a significant effect of inducing lipolysis (*p* < 0.05 compared to negative control). With respect to the controls (positive and negative), they are statistically significant as well.

### 3.2. Effects of Bean Extracts on Cytotoxicity

**Figure 2:**
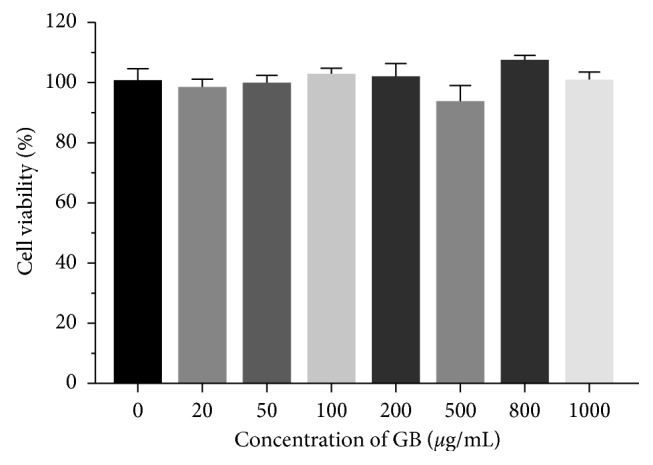
Effect of green bean extract on mitochondrial dehydrogenase activity. 3T3-L1 preadipocytes were incubated for 48 hours with concentrations of 20 to 1000 *μ*g/mL of green beans (GB). MTT values were calculated as a percentage respect to untreated cells. *n*=5 in each assay.

Before assaying the extracts with the cell line, it was necessary to carry out a cytotoxicity test to obtain the concentration at which the extract begins to be cytotoxic, in order to rule out that the extract does not influence cell viability. The level of viability that was required should be >90%. A cell viability curve was performed in a range of 20 to 1000 *μ*g/mL of green bean concentrations. To investigate whether *P. vulgaris* exhibits cytotoxicity, mitochondrial dehydrogenase was assessed using the MTT assay. None of the concentrations of green bean extracts used during the study decreased mitochondrial dehydrogenase activity, as shown in Figure 2.

### 3.3. Effects of Bean Extracts on Lipolysis *In Vitro*

**Figure 3:**
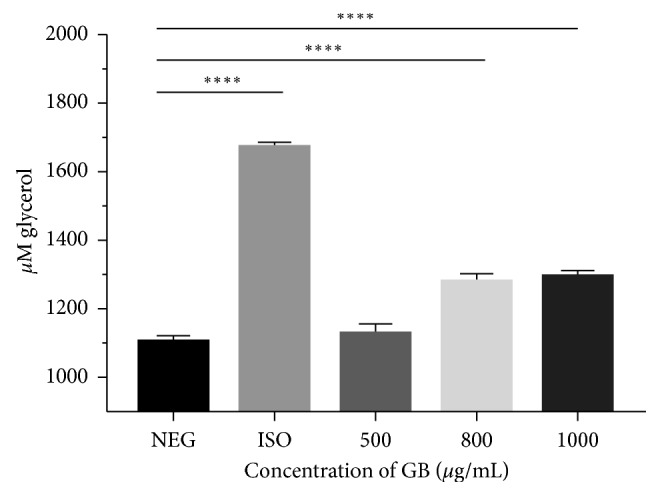
Lipolytic activity of green bean extract on 3T3-L1 mature adipocytes. NEG: negative control; ISO: positive control of 10 *μ*g/mL isoproterenol and green bean extract (GB); *n*=8. ^*∗∗∗∗*^*p* < 0.0001.

Treatment of 3T3-L1 adipocytes with the extracts and isoproterenol, a nonspecific adrenergic agonist, increased the amount of glycerol released (Figure 3) although statistical significance was noted for green bean extract concentrations between 800 and 1000 *µ*g/ml induced lipolysis, with maximal stimulation observed from 800 *µ*g/ml (*p* < 0.001).

### 3.4. Effects of Bean Extract on Lipid Droplet Formation in 3T3-L1 Adipocytes

**Figure 4:**
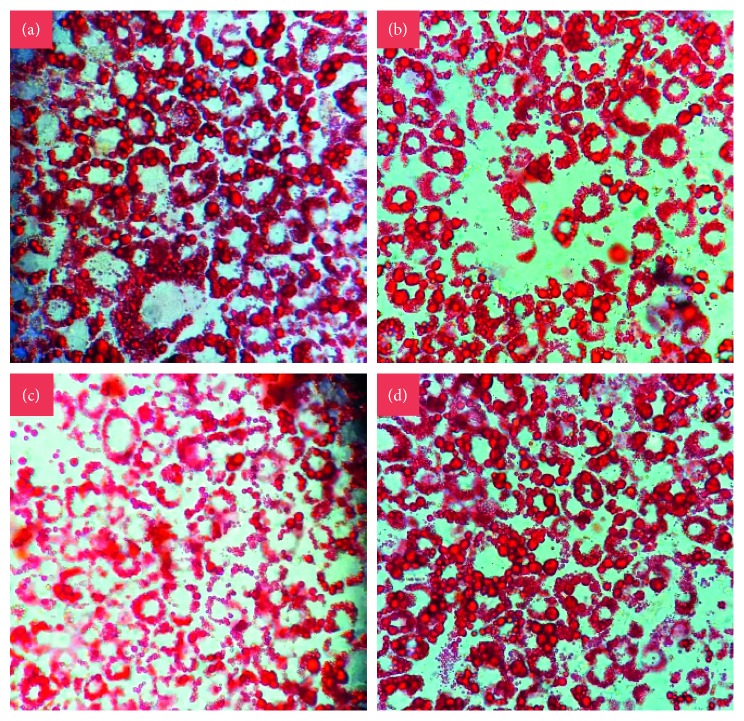
Staining with Oil Red O. Effect of green bean extract on adipogenesis of 3T3-L1 cells. (a) Mature adipocytes stained on Day 8 after induction of differentiation. Mature adipocytes stained were subjected to differentiation with 500 *μ*g/mL (b), 800 *μ*g/mL (c), and 1000 *μ*g/mL (d) of GB, on Day 8 after induction of differentiation. 40x objective photographs.

The differentiation of preadipocytes to mature adipocytes is associated with the increase in the number of cells stained with Oil Red O and lipid accumulation. The microscopic observation of the Oil Red O stain shows an intracellular reduction in the number and size of lipid droplets accumulated in the cells treated with GB at the three concentrations compared to the control cells or those that were not treated and that reached complete differentiation (Figure 4).

**Figure 5:**
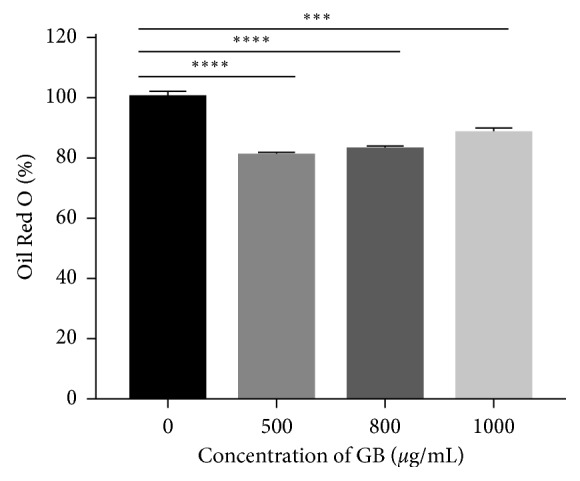
Green bean extract inhibits the intracellular accumulation of lipids in 3T3-L1 cells. The values of total lipid accumulation relative to spectrophotometry of the staining (492 nm) were calculated as a percentage relative to the untreated cells. *n*=5. ^*∗∗∗*^*p*=0.0006; ^*∗∗∗∗*^*p* < 0.0001.

Next, the accumulation of fat after cell differentiation of treated and untreated cells with green bean was quantified spectrophotometrically. The different concentrations of green bean extract exhibited similar effects on lipid accumulation, as shown in Figure 5. Concentrations of 500 to 1000 *µ*g/mL reduced intracellular lipid accumulation by 20% compared to control (*p* < 0.001).

### 3.5. Effects of Extracts on Intracellular Triglyceride (TG) Content in 3T3-L1 Adipocytes

**Figure 6:**
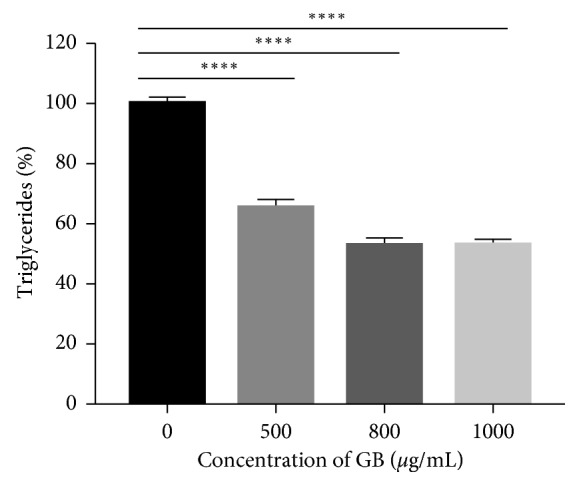
Green bean extract inhibits the accumulation of triglycerides in 3T3-L1 cells. The reduction of the total triglycerides per well was calculated as a percentage relative to the untreated cells. *n*=8. ^*∗∗∗∗*^*p* < 0.0001.

Figure 6 shows that all concentrations of the green bean extract (500 to 1000 *µ*g/mL) decreased intracellular triglyceride accumulation compared to the untreated control. Green bean extracts reduced triglyceride accumulation by 34.4% at 500 *µ*g/mL and 47.1% at 800 and 1000 *µ*g/mL (*p* < 0.0001).

## 4. Discussion

The high mortality presented by CVD is directly related to the high prevalence in the population of modifiable cardiovascular risk factors such as diet [19]. It is therefore necessary to create a “new nutrition” that relates the healthy diet as a key factor in health and quality of life. It is therefore essential to promote a healthy lifestyle and diet in the population and to encourage the consumption of a Mediterranean diet, which is rich in fruits and vegetables.

Therefore, plant foods such as fruits and vegetables, in addition to the nutritional contribution, may be used as healthy beneficial elements such as functional foods [20] and bioactive phytochemicals. Another important risk factor in the development of CVD and ischemic events is obesity [21]. Until recently, it was thought that adipose tissue was exclusively involved in energy reserve, but now, there is enough evidence showing that adipose cells exert autocrine, paracrine, and endocrine effects [22]. The excessive accumulation of fat in different parts of the body is a risk factor for the secretion of inflammatory mediators and hormones that regulate metabolism and intake, including cytokines such as TNF-alpha and IL-2, key inflammatory mediators in endothelial dysfunction, the first event in the development of CVD [23]. Our aim was to evaluate the ability of bean extracts to induce direct lipolysis in adipose tissue. This effect has not been reported for these beans, and the results clearly show that the bean extracts significantly stimulated lipolysis in rat adipose tissue *ex vivo*. Along with this, we have determined that the compound or compounds responsible for the observed effect is modulated by the state of maturation of the grain, being green bean extract that presented a stronger effect than shelled bean extract. Moreover, it is probably that the responsible molecule for this activity has a higher partition in the aqueous solvent since greater effect can be observed in the aqueous than in the methanolic extracts. There has been a recent study which evaluated the ability of extracts of “black adzuki bean” on adipocytes in culture and found that this extract has antiadipogenic effects [24]. Another recent publication by Stefanon et al. showed that extracts of *Rosmarinus officinalis* can modulate adipocyte differentiation and metabolism, modulating triglyceride accumulation [25].

Most of the studies looking for effects at the level of adipose tissue using extracts are based on diets with these natural products. With the use of an *in vitro* model of adipocyte cells, we have focused on testing the effect directly on preadipocytes and mature adipocytes in cell culture conditions and therefore assessed their lipolytic and inhibitory effects on adipogenesis.

In relation to obesity and in search for new natural strategies for its treatment, our objective was to evaluate the ability of green bean extract to induce lipolysis directly on mature 3T3-L1 adipocytes *in vitro*, an effect that has not been reported by this type of bean in a cell culture model. After incubation for 48 hours with the extract, our results indicate that, at concentrations of 20, 40 and 60 *μ*g/ml, there is no increase in glycerol in the extracellular medium; therefore, direct stimulation of lipolysis was not reported. With these results, we discarded the use of low concentrations due to its limited effect; however, in previous studies with green bean, this extract at a concentration of 1 mg/mL presents direct lipolytic activity on rat white adipose tissue, an *ex vivo* model, in comparison with aqueous and methanolic garnet bean extracts that did not have significant results on direct lipolysis of the same model.

Although in the *ex vivo* model, we used this concentration of extract, incubated for 1 hour on adipose tissue, we decided to submit 3T3-L1 cells to concentrations of 1000 *μ*g/mL in the cell viability experiment. Our results indicate that higher concentrations of the extract do not cause negative effects on the cells because after incubation, they maintain viability greater than 90%. With this, we decided to carry out the same *in vitro* lipolysis experiment with green bean extract but with concentrations of 500, 800, and 1000 *μ*g/mL, respectively. The results indicate that, at concentrations of 800 and 1000 *μ*g/mL, there is direct stimulation of lipolysis. In addition, it is shown that there is a tendency that the release of glycerol is increased as the concentration increases, evidencing that these types of bean have lipolytic activity both *in vitro* and *ex vivo*. In the literature, this phenomenon has been related to the high polyphenol content of *Phaseolus vulgaris* (0.4%), where the main polyphenolic compounds correspond to tannins, phenolic acids, and flavonoids. Among the vegetables with the highest content of polyphenols are pigmented beans such as the red bean (*P. vulgaris*) and the black bean (*Vigna mungo*), noting that these bioactive compounds have important antioxidant properties [26, 27].

In most studies where there is an implication effect of beans, the experiments are based on the intake of *P. vulgaris*, with a reduction of body mass, satiety, and lipids in obese animals [28]. A series of inhibitory proteins of hydrolases present in beans are active against proteases, amylases, lipases, and glycosidases. It has been shown that the administration of an inhibitor derived from the black bean slows the digestion of starch with the reduction of serum glucose and insulin concentrations, in addition to increasing the metabolism of nonesterified fatty acids from adipose tissue in rats. The extract of *P. vulgaris* inhibits the activity of *α*-amylase, preventing the enzyme from acting on starch, and therefore, the postprandial hyperglycemia is reduced, which leads to less fat storage in adipocytes [15, 29]. Another compound found in beans is lectins, specifically phytohaemagglutinin, a glycoprotein associated with the reduction of insulin levels and lipid accumulation [30]. Therefore, one way in which compounds induce low body mass is indirectly through satiety and causing a low starch digestion. Our results propose that bioactive compounds of green beans also exert a direct mechanism on adipocytes through lipolysis.

Related to the aforementioned, *in vivo* studies have reported that the daily use of an oral nutritional supplement with 445 mg of an extract of *P. vulgaris* (“Phase2TM”), derived from white bean, during 30 days in overweight volunteers, caused a significant decrease in body mass and decrease in fat mass. Therefore, the inclusion of *P. vulgaris* in the diet and/or *in vitro* studies on adipocytes is an interesting antiobesity target [31].

There is a series of natural compounds in fruits and vegetables with lipolytic effects, but those associated with beans are few. However, in 2012, a study evaluated the effect of “black adzuki” (*Vigna angularis*), a legume grown in the east, as a potential antiobesity product. In this study, it is indicated that this extract exerts a significant reduction in the accumulation of liver lipids in *in vivo* studies. On the contrary, in a *in vitro* study, incubation of human adipocytes with the extract (250, 500, and 1000 *μ*g/ml in sterile water) produced a significant decrease in triglyceride accumulation without affecting the cell viability and the reduction of inflammatory response [32]. Although in this work intracellular triglycerides were not quantified in mature adipocytes submitted to the GB extract, glycerol levels were measured to determine lipolysis. It is expected that there may also be an intracellular fat reduction; our results are comparable since they used concentrations higher than 0.5 mg/ml.

It has recently been published that black adzuki bean has antiadipogenic effects in adipocyte culture. In this study, it was observed that the extract is able to inhibit cell proliferation and suppress adipogenesis in early phases of differentiation associated to a lower expression of C/EBP*β*. In addition, it causes a reduction of triglycerides depending on dose and time; there was also lower expression of GLUT4, FABP4, LPL, and adiponectin. Compared to the control group, the groups treated with bean extract exhibit a higher expression of lipolytic genes such as ATGL and HSL [24]. Regarding our results and the effects on adipogenesis of 3T3-L1 preadipocytes until mature adipocytes, they indicate that there is a reduction in lipid accumulation at the end of the test, as well as triglycerides, suggesting that the green bean exerts an anti-adipogenic effect. The concentrations used are also high and range from 500 *μ*g/mL to 4 mg/mL and from 0.5 to 2.5 mg/mL; the extract of the adzuki bean is not toxic as well as the green bean, which suggests that beans in general can be used at these concentrations on average, when used in adipocyte cell cultures. Unlike the study indicated above, we cannot determine whether the green bean exerts inhibition of early or late adipogenesis because we did not quantify the expression of genes such as PPAR*γ* and C/EBP *α* and *β*. It should be noted that the increase in HSL expression, lipolysis itself, also indicates the possible way in which the green bean acts on mature adipocytes; therefore, it may also be important to measure the expression of this gene in adipocytes.

As mentioned at the beginning, beans in general and especially the pigmented ones contain several polyphenols such as proanthocyanidins, quercetin, and genistein. These antioxidants have been shown to inhibit proliferation and adipogenesis. Other studies suggest that genistein promotes lipolysis and inhibits adipogenesis in cell culture, in addition to the fat content of preadipocytes in differentiation [33]. This suggests that the polyphenolic compounds of the green bean extract are those that exert the lipolytic and antiadipogenic effects shown in the results; therefore, a projection is to evaluate and quantify the content of antioxidants and polyphenols of the extract itself.

Another compound with antioxidant properties that has been described in a series of vegetables, fruits, and tea is myricetin, a flavonoid. This flavone has been described as anticarcinogenic, anti-inflammatory, and antihyperlipidemic; however, it was recently described that myricetin suppresses the differentiation of 3T3-L1 preadipocytes by reducing the expression of C/EBP*α* and PPAR*γ* together stimulates lipolysis in adipocytes by reducing the expression of perilipin A, classifying it as an antiobesity compound [34]. The latter also suggests that green beans may not only induce lipolysis by increasing the expression of HSL but also by stimulation of pathways related to perilipin A; it can also have an effect on cell differentiation by inhibiting the expression of genes C/EBP*α* and PPAR*γ*.

The lipolytic and antiadipogenic effects arising from bean extracts represent an interesting food strategy in the treatment of cardiovascular diseases because it directly affects one of the most important risk factors for the development of CVD and obesity. This study is a starting point for identifying and studying the active principles and effects *in vivo*.

## 5. Conclusion

Here, we showed that green bean extract has a direct lipolytic effect on mature adipocytes 3T3-L1, with the consequent release of glycerol, at concentrations of the extract that do not affect cell viability. The use of the green bean extract from the beginning of preadipocyte differentiation shows antiadipogenic effects; therefore it reduces lipid accumulation at the end of the process.

## Data Availability

The data used to support the findings of this study are available from the corresponding author upon request.

## Abbreviations

IBMX: 3-Isobutyl-1-methylxanthine
MTT: 3-(4,5-Dimethylthiazol-2-yl)-2,5-diphenyltetrazolium bromide
CVD: Cardiovascular diseases
TNF: Tumor necrosis factor
GB: Green bean
C/EBP: CCAAT-enhancer-binding proteins
GLUT4: Glucose transporter type 4
FABP4: Fatty acid-binding protein 4
LPL: Lipoprotein lipase
ATGL: Adipose triglyceride lipase
HSL: Hormone-sensitive lipase
PPAR: Peroxisome proliferator-activated receptor.
